# Unmet challenges in septoplasty–nordic studies from a uniform healthcare and geographical area

**DOI:** 10.3389/fsurg.2022.1061440

**Published:** 2022-12-01

**Authors:** J Hellgren, M Lundberg, N Rubek, C von Buchwald, S Steinsvåg, A Mäkitie

**Affiliations:** ^1^ Department of ORL, Head & Neck Surgery, Institute for Clinical Sciences, Sahlgrenska Academy, University of Gothenburg, Gothenburg, Sweden; ^2^ Department of ORL, Head & Neck Surgery, Region Västra Götaland, Sahlgrenska University Hospital, Gothenburg, Sweden; ^3^ Department of Otorhinolaryngology—Head and Neck Surgery, University of Helsinki and Helsinki University Hospital, Helsinki, Finland; ^4^ Department of Clinical Allergy and Immunology, Brigham and Women’s Hospital, Harvard Medical School, Boston, MA, United States; ^5^ Department of Otorhinolaryngology—Head and Neck Surgery and Audiology, Rigshospitalet, Copenhagen, Denmark; ^6^ Department of ORL Head & Neck Surgery, Sørlandet Hospital, Kristiansand, Norway; ^7^ Department of ORL Head & Neck Surgery, Haukeland University Hospital, Bergen, Norway

**Keywords:** PROM (patient reported outcome measures), septoplasty outcomes, quality register, nordic, review

## Abstract

**Purpose:**

Nasal septoplasty is one of the most common surgical procedures in otorhinolaryngology and optimising both patient selection and the surgery is a challenge. The Nordic countries have similar public healthcare systems and comparable populations in terms of size.

**Methods:**

This is a review of studies of outcome and predictors related to septoplasty from Denmark, Finland, Norway and Sweden, published during the last decade. The aim of this review was to identify areas in need of further research to meet the challenges of septoplasty in the Nordic countries with reference to international data.

**Results:**

Postoperative patient satisfaction at 6–12 months was reported in around 2/3 of the patients and well in line with international data. Patients with more severe symptoms had a higher chance of improvement. Lack of standardisation in patient selection, surgical methods and skills, and follow up procedures, still makes it difficult to explain the 25% failure rate in septoplasty surgery.

**Conclusion:**

This review of the Nordic studies from the last decade shows that septoplasty in general is effective in relieving nasal obstruction. There is a need for studies addressing the standardisation of diagnostic tools and algorithms and the systematic and continuous implementation of follow-up of the surgical results at both departmental and personal level. This includes an awareness of how surgical skills in septoplasty are obtained and maintained.

## Introduction

Septoplasty is a common surgical procedure in the field of Otorhinolaryngology—Head and Neck Surgery (ORL-HNS). The main indications address functional deficits caused by a deviated nasal septum, which can usually be corrected satisfactorily. Septoplasty has been part of the ORL-HNS practice for more than 100 years and most of the basic principles were developed long before evidenced medicine was introduced. Numerous non-randomised studies support the clinical benefits of septoplasty, but, only recently, the first randomised study by van Egmond et al. supported that septoplasty was more effective than conservative measures in managing a deviated septum (1). In studies where objective measurements of nasal airflow and resistance are used pre- and postoperatively, the results often confirm that a significant improvement in objective measurements has really been achieved after surgery (2). In a comprehensive review of septoplasties from different parts of the world published in 2018, patient satisfaction after surgery (measured as patient-reported outcome measures, PROMS) was reported to be between 50% and 100% after a minimum of 12 months postoperatively, indicating scope for improvement (3). The correlation between patients' expectations, PROMS and objective measurements of nasal patency also demonstrates conflicting results (4, 5). It is therefore crucial that the expected surgical outcome is considered carefully and discussed with the patient in the light of the known risk of complications, including an unsatisfactory surgical result (5, 6).

Areas of concern contributing to successful septoplasty service include surgical indications and patient selection, preoperative measurements (acoustic rhinometry, rhinomanometry, peak nasal inspiratory flow (PNIF)), PROMs, surgical technique and skills, perioperative considerations (use of antibiotics, local vs. general anaesthesia), postoperative care (day-care surgery vs. postoperative hospital stay) and evaluations of surgical management (including quality of life studies). It remains a challenge to study these factors separately and systematically and to compare the results between healthcare systems and surgical centres.

The Nordic countries, Denmark, Norway, Finland and Sweden, are all affluent countries with a governmental tax-financed public healthcare system for all citizens. Most patients undergoing septoplasty are diagnosed and operated on at ORL-HNS departments and by publicly employed surgeons without any personal reimbursement related to the number or type of surgeries performed. The indications for septoplasty are regulated to manage functional upper airway obstruction and, to a minor extent, to facilitate FESS surgery, the use of nasal CPAP masks, in tumour surgery or to treat nasal trauma. The private sector is comparatively small except in Denmark and regulated by public healthcare contracts and clinical guidelines and the indications are thus similar to those in the public system. In Denmark, around one third of simple rhinology is treated in the private sector, including septoplasty, turbinate reduction and other types of surgery which do not require postoperative hospitalisation. However, during the last two years, i.e., during the current Covid pandemic, more than 90% of the septoplasties have been performed in the private sector. Specific differences regarding the use of local anaesthesia, preoperative antibiotics, diagnostic rhinomanometry or other diagnostic objective measurements and surgical techniques still exist between the Nordic countries, as well as between individual centres. In Sweden, the annual number of septoplasties is around 20/100,000, Denmark 48/100,000 and Finland 27/100,000.

During the past decade, several studies from the Nordic countries have addressed the aforementioned factors related to the successful outcome of septoplasty. The data in the Nordic studies are derived either from conventional clinical cohorts or from the unique Swedish national septoplasty registry (SNSR). The SNSR holds limited yet important data regarding indication, surgical procedure and pre- and post-op symptoms of septoplasties from an entire country since 1997 (https://sep.registercentrum.se/omregistret/septumplastikregistret/p/Sk0aanMH).

In this article, we discuss the Nordic experience of septoplasty in terms of surgical indications, objective outcome parameters and PROMS. The purpose is to suggest areas for further research to improve the quality of the outcome of septoplasty surgery.

## Indication for septoplasty

The main indication for septoplasty according to the American Academy of Otorhinolaryngology, Head & Neck Surgery, is “Nasal airway obstruction (unilateral or bilateral) causing any of the following: mouth breathing, snoring, nasal congestion, sleep apnea, unresponsive to medical management” (https://www.entnet.org/resource/clinical-indicators-septoplasty/). This also relates well to the Nordic situation, apart from snoring and sleep apnea not being primarily treated by septoplasty alone. In 2019, 2,138 septoplasties with or without turbinoplasty were performed in Sweden, according to the National Patient Register (PAR, https://www.socialstyrelsen.se/en/statistics-and-data/registers/register-information/the-national-patient-register/), where all surgical procedures in Sweden are registered. Sixty per cent (1,291) of these were also reported to the SNSR, which includes only septoplasty with and without turbinoplasty. In 2019, 52% of the procedures in the SNSR were septoplasties alone and 48% were septoplasties combined with a turbinoplasty.

## The severity of nasal obstruction

In the SNSR, the patient-reported subjective nasal obstruction is rated as none, mild, moderate, or severe before and 12 months after surgery. In a study of 888 patients from the SNSR operated on in 2015–2016, 63% of the patients reported an improvement in their nasal obstruction 12 months after septoplasty (7). This result is in line with the previously presented international data from the review by Tsang et al. (3). When examining the data from the SNSR more closely, the improvement in nasal obstruction after septoplasty was related to the severity of nasal obstruction before surgery. In patients with severe nasal obstruction before surgery, 81% had improved compared with patients with moderate (57%) and mild nasal obstruction before surgery (31%). This was also seen in a study from Finland where septoplasty alone was performed on 188 patients (mean age 41) (8). In this study, the patients were evaluated by the Sino-Nasal Outcome Test 22 (SNOT-22). While the item of nasal blockage decreased by 46% in the whole study group after septoplasty, a negative effect was seen on the health-related quality of life (HR-QoL) in patients with mild symptoms preoperatively. Previous data, such as the study by Stewart et al., support the findings that patients with worse symptoms experience a greater improvement after septoplasty (9).

## Preoperative clinical evaluation

Rhinoscopic evaluation by a trained and experienced nasal surgeon is a prerequisite in all decision-making relating to whether or not to perform a septoplasty (10). However, the benefit of using additional objective assessment tools such as rhinomanometry, peak nasal inspiratory flow (PNIF) or acoustic rhinometry has been debated (11). According to the SNSR in 2018 and 2019, around 60% of the surgical centres in Sweden applied rhinometry as an integrated part of the preoperative work-up and the systematic use of rhinomanometry has also been applied in Finland, but not in Denmark. As yet, there has been no comparison between the centres in the SNSR that use preoperative rhinomanometry and the centres that do not, nor has this been done in Finland. However, in overall terms, the Nordic results are not superior to those in other international studies without a preoperative rhinomanometric evaluation. If performed before and after decongestion, rhinomanometry offers an opportunity to diagnose significant inflammatory mucosal disease. It can also reveal “sensory” nasal obstruction where nasal airflow is not restricted, but the patient still perceives a blocked nose. As yet, the scientific support for its regular clinical use in relation to the additional labour and cost involved is not convincing. In spite of this, its use in the Nordic setting has been justified by its properties that support the surgeon to decline surgery when the indication is weak and thus potentially avoid unnecessary surgery. A Danish study addressing the prognostic value of preoperative acoustic rhinometry in septoplasty, in combination with other nasal surgery, showed that 56% of 222 patients were satisfied with the surgical outcome when interviewed 11 years (mean follow-up time) after surgery. Satisfaction was associated with three-month postoperative acoustic rhinometry improvements, but it was concluded that acoustic rhinometry did not show preoperative patient selection potential (12).

A few other recent Nordic studies have evaluated the effect of septoplasty with and without turbinoplasty on nasal airflow resistance and intra-nasal geometry. In a study from Norway, 148 patients were investigated pre-operatively and six months postoperatively in a prospective, observational design. Fifty patients underwent septoplasty, 51 underwent septoplasty combined with radiofrequency turbinoplasty and 47 underwent radiofrequency turbinoplasty alone (13). The minimal cross-sectional area (MCA) and the nasal-cavity volume (NCV) measured with acoustic rhinometry improved significantly in all three groups. The MCA 0–3 cm from the nostril increased by 11% in the septoplasty group and by 7% in the septoplasty + radiofrequency turbinoplasty group. PNIF improved in all three patient groups by 36%–40%, confirming that surgery produced an improvement in nasal patency. Increased nasal geometry correlated with reduced nasal obstruction measured on a Visual Analogue Scale (VAS).

Another study from Norway investigated patients, exploring the use of PNIF (unilateral, bilateral and combined unilateral (left + right)) to evaluate the results of septoplasty alone (*n* = 58) and septoplasty with turbinoplasty (*n* = 68) (14). PNIF increased significantly by 23% in the septoplasty only group and by 38% in the septoplasty + turbinoplasty group. Additional turbinoplasty thus resulted in a better outcome than septoplasty alone. The VAS for nasal obstruction in the whole group improved by 67%. Objective measurements of nasal patency thus confirmed the improvements made by septoplasty, as has been reported previously, but not all patients experience this improvement (2).

## Surgical skills

Surgical skills remain fundamental to a successful septoplasty outcome. Surgical training programmes on how to perform septoplasty follow a similar track in the Nordic countries. Specialist training mainly takes place in hospital ENT clinics where surgical skills are gradually transferred from senior to junior doctors in training, primarily through four-handed procedures. Doctors in training first perform surgery assisted or observed by an experienced colleague until they are considered autonomous. The UEMS logbook is used by residents in training (www.orluems.com). In the Nordic countries, the ability to perform uncomplicated septoplasty and turbinate reduction is a requirement to acquire specialist training certification, though the level of autonomy vary between the countries.

In the study of 188 septoplasties from Finland, the surgeon's experience was evaluated in relation to the surgical result for the disease specific HR-QoL instrument SNOT 22, but it was not found to be a significant factor (8). Another study from Finland including 76 septoplasties operated by specialists (71%) and residents (29%), found that being operated by a senior surgeon was associated with a better SNOT 22 result, twelve months after surgery, (adjusted OR 9.9, 95% CI 1.5–67) (15). In a study from the SNSR, it was shown that patients undergoing surgery at university clinics were significantly less satisfied with the surgery after six months than the patients undergoing surgery at county hospitals (16). This may be somewhat surprising, since surgical skills could be expected to be high at university clinics. However, at university clinics, surgical training is more frequent and revision surgery and challenging operations are more common than in county hospitals.

Even though explicit surgical skills were poorly assessed in the Nordic studies, the varying number of involved surgeons could indirectly reflect varying skills in these series. The number of surgeons ranged from one to several hundred in the SNRS studies. The overall postoperative result in terms of patient satisfaction after surgery was, however, surprisingly similar. This could be an indirect sign that surgical quality is maintained at centres with less experienced surgeons under training, by a system for surgical supervision and surgical aid for the less experienced. In a study from the SNRS based on 5,865 septoplasties over a 10-year period, including only clinics with a reporting rate of >70% of all surgeries performed, 76% of the patients reported total or near total symptom relief after six months (11). This is almost identical to the results from an earlier Finnish study, where 75% of 117 septoplasty patients reported no or mild nasal obstruction one year after surgery (17). In that latter study, 80% of the surgeons were specialists and 20% were residents.

A collaborative study between Norway and Sweden evaluated pre- and postoperative nasal obstruction with a VAS for nasal symptoms in 366 consecutive patients operated on by a single experienced ENT surgeon (18). The study comprised 159 patients undergoing septoplasty alone, 79 septoplasties combined with turbinoplasty and 128 turbinoplasty alone. In the septoplasty-alone group, the VAS for nasal obstruction improved by 62% and, in the septoplasty + turbinoplasty group, it improved by 67% after three to six months. In a study with a similar design, from a university clinic in Norway, 171 patients were operated on by 14 different surgeons (19). The mean VAS for nasal obstruction six months after surgery improved by 61%, well in accordance with the results from the single-surgeon study. The patients who underwent septoplasty combined with radiofrequency turbinoplasty reported less nasal obstruction postoperatively than patients who only underwent radiofrequency turbinoplasty.

## Health-related quality of life

SNOT-22 has been used in several Nordic septoplasty studies to assess HR-QoL before and after surgery, although it was primarily designed for chronic rhinosinusitis and not for disease caused by a deviated septum. In a study from Finland, 76 patients underwent septoplasty and were assessed with SNOT-22 before and 12 months after surgery. The score improved by 15 points on average, and in 64% of the patients there was an improvement of more than 9 points, the minimally important difference defined for chronic rhinosinusitis (CRS) (15). In the study of 171 patients from Norway, the patients operated with septoplasty combined with radiofrequency turbinoplasty had a mean improvement in their SNOT 20 of 0.8, six months after surgery, considered the minimally clinical difference in CRS (19). In the study of 188 patients from Finland, the mean SNOT-22 of 21.52 before surgery was reduced to 17.40, six months after surgery, an improvement of 4.1 points (8). The symptom of “nasal obstruction” in the SNOT-22 questionnaire decreased from 2.96 before surgery to 1.60, making a difference of 1.36 (46%).

## Long-term results

Studies of septoplasty results exceeding a follow-up of 12 months are scarce (3). In this Nordic review, we found two studies. The study from Denmark, in which 222 patients were interviewed by telephone, showed a 56% satisfaction rate at a mean follow-up of 11 years after surgery (12). In one long-term follow-up study from the SNRS comprising 111 patients from one county hospital in Sweden, 53% had remaining or worse symptoms at follow-up 30 to 70 months after surgery (20). This indicates that the result after septoplasty may decline with time, as was also found in the review by Tsang et al. (3).

## Other factors that could affect the surgical outcome

The age of the patients at surgery has been studied in two Nordic studies. In the study from the SNSR comprising 5,865 patients (mean age 39 years), higher patient age was associated with a significantly better result measured as self-reported patient satisfaction, six months after surgery (16). On the other hand, in the study from Finland comprising 188 patients (mean age 41 years), higher age was associated with a poorer result (8). This has also been reported previously, but the impact of patient age on the outcome still remains unclear (21).

In the SNSR, postoperative unplanned visits due to infection, bleeding or pain were related to a significantly poorer outcome regarding both patient satisfaction and improvement in nasal obstruction (7, 16). Bleeding and infection in septoplasty with or without turbinoplasty were reported to be around 3% in a large study from Poland (6). In a recent Finnish study, the number of postoperative infections were reduced from 6.1% when no antibiotics were administered to 2.6% with one dose of prophylactic cephuroxime (22). Preoperative antibiotics in septoplasty is, however, not a standard practice in the Nordic countries.

In recent years an increasing interest has been directed towards the significance of trigeminal function and the ability to lateralize the perception of menthol exposure to either of the two nasal sides (23–25). It has been suggested that the preoperative evaluation of the trigeminal sensitivity could improve over-all patient satisfaction after septoplasty. In this Nordic review we found no studies addressing this interesting topic.

## Discussion

The aim of this review, targeting the outcome of septoplasty surgery in the Nordic countries over the past decade, was to define scientific questions that remain unanswered and to compare the Nordic setting with global surgical data. It is obvious that septoplasty is a relevant surgical procedure relieving chronic nasal obstruction in at least 2/3 of the patients and improving their health-related quality of life.

We have focused on septoplasty with and without turbinoplasty with the indication nasal obstruction, leaving out septoplasty to facilitate FESS or as an emergency procedure in trauma. In all the reviewed studies, the indication for septoplasty was established by nasal surgeons examining the patients and taking a patient history, sometimes with the aid of rhinomanometry. The lack of standardisation in these procedures is a problem when it comes to monitoring indications and outcome in septoplasty, but it can also be seen as an expression of the substantial difficulties associated with objective and subjective assessments of nasal airflow and resistance. Clinical decision-making and patient selection for septoplasty surgery clearly warrant uniform guidelines in order to enable an evaluation of postoperative results in a multicentre setting.

Septoplasty used on a broader scale as a one “fits all” type of surgery for any structural nasal obstruction in accordance with tonsillectomy for tonsillitis is inadequate. Issues related to the nasal valve, columella, or nasal deformities may be totally overlooked. We found no Nordic studies evaluating the technique of a nasal examination using anterior rhinoscopy and nasal endoscopy in the diagnostics of patients undergoing septoplasty or its impact on the result. Nasal endoscopy with a flexible or rigid endoscope is typically performed in the evaluation of a symptomatic septal deviation to assess the full length of the nasal cavity and to exclude concha bullosa, nasal polyps or tumours as the cause of nasal obstruction. Imaging of the nasal cavities such as computer tomography (CT) or Cone Beam CT (CBCT) are not in regular use to evaluate symptomatic septal deviations in the Nordic countries due to radiation exposure and availability. When performing an anterior rhinoscopy, the columella with the most anterior part of the septum is easily pushed aside and therefore ignored. The sensors of nasal openness are in the most anterior part of the nose, in the vestibular area (26). Differences in trigeminal function could affect the surgical result. In a recent study of patients with a symptomatic septal deviation, the nasal heat flux (cooling) and menthol lateralisation test was distorted compared with subjects having a septal deviation without symptoms or no septal deviation at all (27). This implies that interest should primarily focus on anterior abnormalities. The deviation of the anterior margin of the septal cartilage behind the columella can mostly be observed without any instruments. It can be easily corrected under local anaesthesia and, if necessary, combined with the correction of alar deformities or insufficiencies. Specific investigations of the effect of surgical measures to open the vestibular area for the feeling of nasal patency are missing and should be conducted. A previous study from Finland elegantly showed that, when the septum is moved, the cross-sectional area on the compromised side is enlarged in favour of the patent side (17). The question of why symmetry between sides is preferred by many patients, while asymmetry is accepted by others, remains obscure.

In fact, it is more likely that the ORL profession as such has inadequate tools and algorithms to properly diagnose and differentiate the different aspects of impaired nasal airflow. For instance, in the diagnosis of heart failure, using echocardiography, the blood flow in the heart can be studied in real time. If a similar non-invasive technique were available for nasal airflow, it would probably increase our diagnostic accuracy and potentially the surgical results. Computional fluid dynamics (CFD) is an emerging and promising technique to study nasal airflow that may change this situation in the future (27).

One obvious and important factor for the success of septoplasty is surgical skill and experience, although data from studies are less conclusive on this issue (28, 29). The one Nordic study aiming for surgical skill did not support this as an important factor (8). In the reviewed Nordic studies, the number of surgeons ranged from one to several hundred and, surprisingly, the overall results were strikingly similar, also compared with international data (3). Breaking this down to individual surgeons is tempting, but it should be remembered that greater surgical skills are often associated with more challenging cases, making a direct comparison unfair if patient-specific factors are not considered. This could explain the poorer results from university clinics compared with county hospitals found in the SNSR. Studies should therefore include both comparable items related to surgical skill and to surgical difficulty.

The surgical technique naturally has an important effect on the surgical result. Modifications of transfixation incision and unilateral or bilateral submucosal surgery are used in the Nordic countries and endoscopic surgery is used to a minor degree, as well as different postoperative packaging from stents, staples, tubing and gauze to septal sutures and no packaging at all. Nasal packaging from one day to one week after septoplasty has been the dominant postoperative tradition in the Nordic countries, however, recently the use of septal sutures or staples instead of nasal packaging has gradually increased. Removal of nasal packaging including nasal stents is perceived by many patients as traumatic and painful which suggest it should be avoided. The rationale for using nasal packaging despite this fact has been to stabilize the septum during the immediate postoperative period and to avoid the formation of septal hematoma that could jeopardize the final result. The use of various nasal packaging was, however, inadequately described in detail in most of the Nordic studies and different techniques were not compared. For this reason, we have chosen not to discuss this further in this article.

Apart from indications for septoplasty based on clinical findings and a patient history of nasal obstruction as such, an assessment of the severity of the disease is important. In at least two Nordic studies, the improvement in nasal patency was greater in patients experiencing severe nasal obstruction before surgery compared with patients with moderate or mild nasal obstruction, which was previously reported by Stewart et al. (9). In the study from the SNSR, 40% of the patients reported severe nasal obstruction before surgery and so increasing this group of patients in favour of patients with moderate to mild nasal obstruction (13% of the patients) would probably improve the overall result after septoplasty, as the improvement was only 57% and 31% in the latter groups but 81% in the severe group. It is not obvious from the Nordic studies why patients with mild symptoms have undergone septoplasty, but it should be a reason for concern and further evaluation if the patient reports mild symptoms of nasal obstruction before surgery.

In accordance with previous international studies, this Nordic review confirms that objective measurements such as rhinomanometry, acoustic rhinometry and peak nasal flow reveal increased nasal geometry and airflow after septoplasty. Objective methods have previously been compared and appear to correlate well with one another (30). A significant improvement in these measurements may, however, not be clinically relevant. The objective methods are limited, since generally accepted normal values related to age, gender, or body mass index (BMI) are lacking. In the most severe cases of symptomatic septal deviation, the findings are clear (high flow resistance or small cross-sectional area), but the value of objective testing lies typically in a more borderline type of flow restriction. The methods are also labour intensive (rhinomanometry and acoustic rhinometry). The fact that rhinomanometry and PNIF do not assess nasal airflow under normal breathing conditions is an additional flaw that is frequently overlooked. The present well-established, objective methods have contributed significantly to our understanding of nasal obstruction, especially regarding allergy and inflammatory disease, but, for diagnostics and the follow-up of septoplasty, there is a clear need for new and refined, simple-to-use, objective methods and algorithms (27).

PROMS are important tools when studying medical interventions over time. In the studies reviewed here, symptom evaluation, satisfaction rate and HR-QoL instruments were used. Although SNOT-20 and -22 were originally developed for the evaluation of chronic rhinosinusitis (CRS), they have also been used to assess septoplasties (31). As in CRS, a change of 9 points has been considered clinically relevant, which is more easily fulfilled in patients with a higher preoperative SNOT-22 score (31). SNOT-20 and 22 were used in studies from Finland and Norway and showed a significant improvement after surgery. Comparing septoplasty results is, however, challenging, as a myriad of different PROMS are used, but none is as internationally accepted as SNOT-22 is in CRS (3). According to an extensive review by Tsang et al., the NOSE questionnaire appears to be suitable, but to date it has not been translated and validated in all the Nordic languages (3).

Expectations of outcome by both patients and surgeons and how this is communicated is a potentially important factor for the surgical outcome. The Nordic healthcare system is based on “informed consent”, meaning that the patient should be provided with the best available information regarding diagnosis and treatment options, as well as expected outcomes and complications. It is not customary, however, to sign a preoperative agreement, as in some healthcare systems. How the surgical problem is communicated to the patient and how the patient perceives the message from the surgeon has not been well studied, but it could be a potentially important factor for success. In particular, the issue of possible complications of septoplasty appears to have been left out of the patient information (5). This discussion could also include objective and documented preoperative information such as rhinomanometry results.

All types of surgery including septoplasty are associated with potential side-effects and complications. In the SNSR studies, unexpected visits to healthcare due to pain, bleeding or infection within the first postoperative period were associated with a poorer self-reported result 6–12 months after surgery (7, 16). In a retrospective study from Poland including 5639 septoplasties, the complication rate was 3.4%. Excessive bleeding was seen in 3.3% and infection causing delayed healing in 3.1%. Both bleeding and infection may lead to either an early and often painful replacement or removal of nasal packaging. This unplanned manipulation of the surgical field and effects of the hematoma and/or infection may affect the healing process negatively. It also gives the patient a negative impression of a surgical complication having occurred. In a study from England where both ENT surgeons and patients were asked if they addressed these complications prior to surgery it was shown that not all of the most common complications were discussed and furthermore, surgeons and patients do rate their severity differently (5). For example, pain was among the complications not discussed with the patients routinely, which could have a negative impact once it occurs*.* Preventing foreseeable complications and reducing their risk could potentially increase patient satisfaction and improve the quality of surgery. While these complications may indicate insufficient surgical management and could lead to longstanding complications such as synechiae and septal perforation, it is likely that their occurrence also undermines the trust of the patient in the surgery.

## Conclusion

Nasal septoplasty is a common and logical/relevant surgical intervention in patients with a deviated septum and nasal obstruction, with a success rate of around 2/3 in the Nordic countries 6–12 months after surgery. Studies from the Nordic countries during the past decade show that patient selection and the degree of nasal obstruction are correlated to surgical outcome. Using the best available surgical technique and postoperative care aimed at achieving the best result, involving PROMS and avoiding postoperative complications also affect the final result. There is, however, a need for further studies addressing the standardisation of diagnostic tools and algorithms and the systematic and continuous implementation of follow-up of the surgical results at both departmental and personal level. This also includes an awareness of the way surgical skills in septoplasty are obtained and maintained.
